# Spectrum of Cardiac Involvement in COVID-19

**DOI:** 10.7759/cureus.8638

**Published:** 2020-06-15

**Authors:** Gauravpal S Gill, Raymond Vlacancich, Neil Mehta, Mansi Chaturvedi, Alexander Papolos

**Affiliations:** 1 Internal Medicine, MedStar Washington Hospital Center, Washington, DC, USA; 2 Cardiology, MedStar Washington Hospital Center, Washington, DC, USA

**Keywords:** covid-19, myocarditis, cardiogenic shock, case series, extracorporeal membrane oxygenation

## Abstract

Cardiac involvement in coronavirus disease 2019 (COVID-19) commonly accompanies multi-organ system failure with acute respiratory syndrome; however, infrequently myocarditis and pericardial effusions may be isolated, yet fulminant. In this report, we highlight significant variations in cardiac involvement and presentation among patients with COVID-19.

This article reports two cases of fulminant myocarditis in COVID-19 positive patients who presented to our facility with contrasting symptoms, laboratory and imaging findings. A 65-year-old patient A had a more typical presentation including respiratory distress, chest pain, ST-segment elevations on electrocardiogram (EKG), lymphopenia, elevated levels of inflammatory markers and cardiac troponin I. A 34-year-old patient B presented with shortness of breath and chest pain similar to patient A; however, she had isolated cardiac involvement with systolic dysfunction and an acute pericardial effusion causing tamponade physiology. Inflammatory marker and cardiac troponin I levels for patient B were within normal range. Patient A had a rapid progression of multi-organ system failure leading to her death within 24 hours from presentation on maximal inopressor support. Patient B, however, is one of few reported cases of cardiac tamponade and veno-arterial extracorporeal membrane oxygenation (VA-ECMO) use in COVID-19 who underwent pericardiocentesis and was additionally managed with colchicine and steroids, leading to complete recovery in systolic function within three weeks from initial presentation.

Isolated myocardial dysfunction and pericardial effusions in COVID-19 may have catastrophic sequalae even in the absence of elevated biomarkers described in literature. Therefore, early detection and management of cardiac involvement is warranted. Additionally, the role of mechanical circulatory support devices and VA-ECMO in COVID-19 needs further investigation.

## Introduction

Coronavirus disease 2019 (COVID-19) infections continue to rise worldwide with latest World Health Organization situation report indicating spread to more than 5.2 million people. According to one report from Wuhan, China, cardiac manifestations are reported in approximately 20% of all hospitalized patients with COVID-19 [1]. Less is known about the susceptibility and clinical phenotypes among cases with cardiac involvement.

In this article, we report two cases of rapidly progressive fulminant COVID-19 myocarditis with varied symptomatology, physical exam, laboratory and imaging findings.

## Case presentation

**Figure 1:**
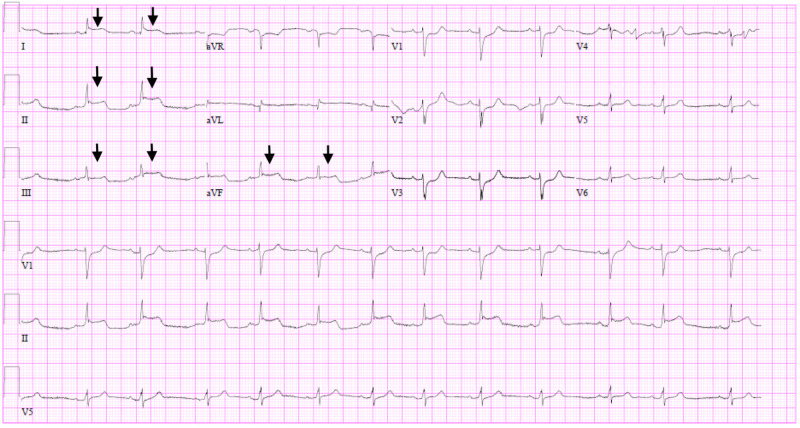
Electrocardiogram depicting ST-segment elevations (arrows) in limb leads (I, II, III, aVF) for Ms. A

Ms. A was a 65-year-old African American woman with a history of breast cancer, diabetes and hypertension who presented with shortness of breath and sharp left-sided chest pain. She had been in contact with her neighbor who was infected with COVID-19. In the emergency department (ED), Ms. A had a temperature of 36 degrees Celsius, a heart rate (HR) of 86 beats per minute (BPM), blood pressure (BP) of 78/58 mmHg and oxygen saturation of (SpO_2_) 94% while breathing ambient air at a respiratory rate (RR) of 25 breaths per minute (Table 1). She had bilateral crackles on auscultation, extremities were cool to touch and her mentation was preserved. Her electrocardiogram (EKG) showed ST-segment elevations in leads I, II, III and aVF with ST-segment depressions in leads V1-4 (Figure 1). Laboratory data were significant for a positive COVID-19 ribonucleic acid polymerase chain reaction (RNA PCR), lymphopenia (500/mL), highly elevated cardiac troponin I (cTnI) at 88 ng/mL and elevated inflammatory markers (Table 1).

**Table 1:** Baseline characteristics, past medical history and diagnostic findings for Ms. A and Ms. B COPD, chronic obstructive pulmonary disease; EKG, electrocardiogram; LVEF, left ventricular ejection fraction

	Reference range	Ms. A	Ms. B
Baseline characteristics			
Gender		Female	Female
Age (years)		65	34
Smoking history		No	No
Past medical history			
Hypertension		Yes	No
Asthma/COPD		No	No
Hyperlipidemia		No	No
Diabetes mellitus		Yes	No
Chronic kidney disease		No	No
Cardiomyopathy		No	No
History of cancer		Yes	No
Admission laboratory findings			
High sensitivity C-reactive protein (mg/L)	<3.00	100.00	1.71
Cardiac troponin I (ng/mL)	<0.045	88.600	0.556
D-dimer (mcg/mL)	<0.50	0.79	<0.27
Creatinine (mg/dL)	0.52-1.04	3.50	0.58
Lactate dehydrogenase (U/L)	84-246	1697	222
Lactic acid (mmol/L)	0.7-2.0	2.3	4.8
White cell count (k/µL)	4.0-10.8	16.2	12.5
Lymphocyte count (k/µL)	0.6-4.9	0.5	2.4
Hemoglobin (g/dL)	11.0-14.5	12.6	15.8
Admission diagnostic findings			
EKG		ST elevations	Low amplitude, PR depressions
Chest X-ray		Bilateral multi-lobar infiltrate	No infiltrate or effusions
Cardiac ultrasound		LVEF=20%	Large pericardial effusion and LVEF=25%

**Figure 2:**
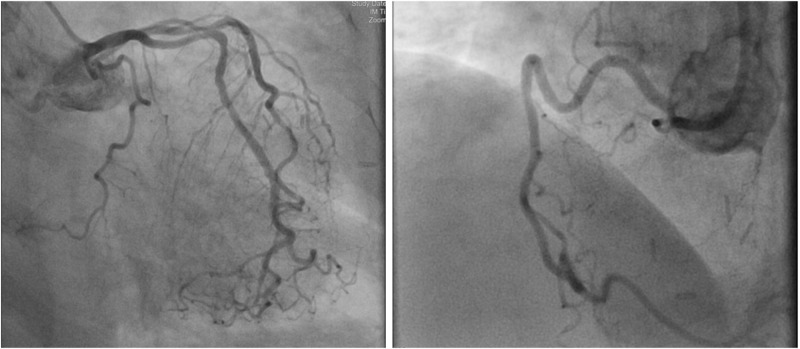
Left and right coronary arteries with minimal luminal irregularities (Patient A)

**Figure 3:**
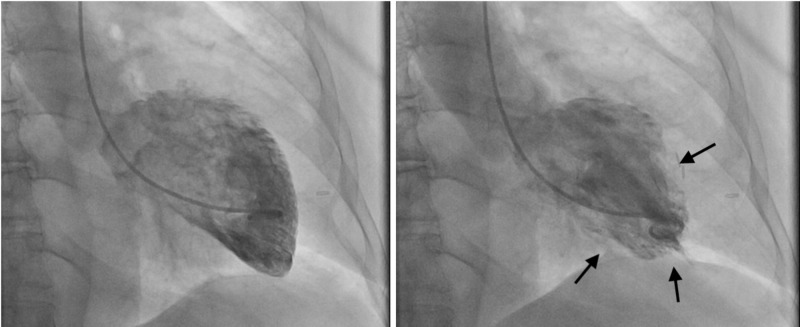
Left ventriculogram at end-diastole (left) and at end-systole (right) for Patient A showing reduced systolic function and arrows indicating wall motion in systole

Coronary angiography and ventriculogram depicted mild luminal irregularities (Figure 2) and a left ventricular ejection fraction (LVEF) of 25% (Figure 3), a significant decrease from the 60% reported by echocardiogram five months prior. Right heart catheterization was notable for a pulmonary capillary wedge pressure (PCWP) of 39 mmHg with a cardiac index of 2.0 L/min/m^2^, after which an intra-aortic balloon pump (IABP) was placed.

**Figure 4:**
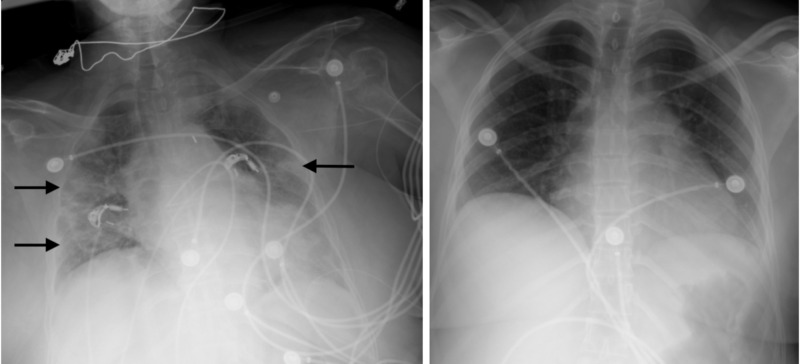
Chest x-ray on presentation for Ms. A (left) depicting diffuse bilateral infiltrate (arrows) vs. chest x-ray on presentation for Ms. B (right) without any significant findings

Her chest x-ray showed diffuse bilateral multilobar airspace opacities (Figure 4). Over the following hours, Ms. A became progressively hypoxic and hypotensive requiring intubation and escalating inotropic and vasoactive support. Her cTnI and high-sensitivity C-reactive peptide (hsCRP) levels increased to >200 ng/mL and 119 mg/L, respectively. Her cardiac index fell to 1.2 L/min/m^2^ despite IABP and four high-dose inopressors. In the context of an institutional scarcity of available mechanical circulatory support (MCS) resources amidst this pandemic, as well as her age, history of diabetes and breast cancer, escalation of MCS to extracorporeal membrane oxygenation (ECMO) was not pursued [2,3]. Ms. A ultimately passed away approximately 24 hours after presentation. 

**Figure 5:**
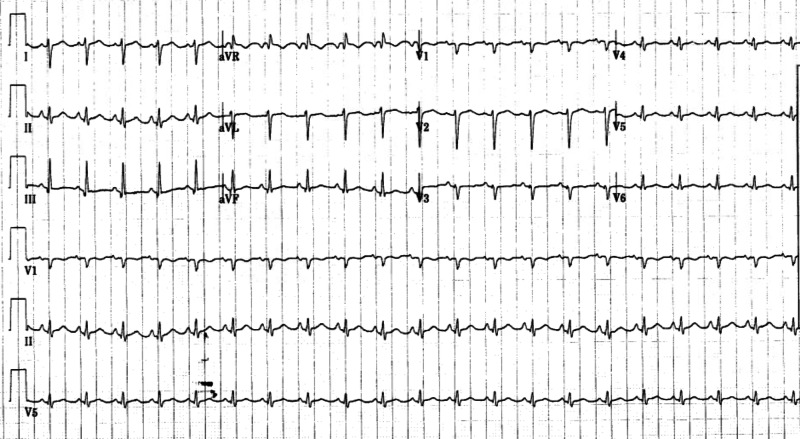
Patient B's electrocardiograms on day prior to admission

**Figure 6:**
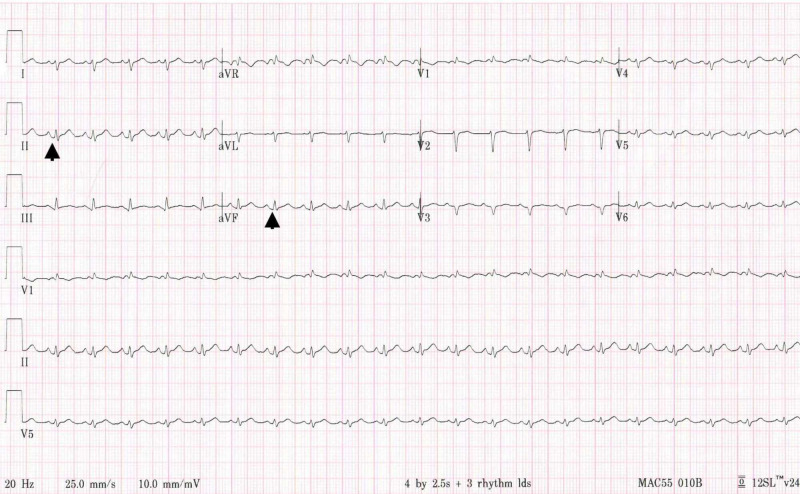
Patient B's electrocardiogram on day of admission showing a decrement in voltage of QRS complexes in both precordial and limb leads with PR depressions (arrows)

Ms. B is a 34-year-old previously healthy Hispanic woman who presented to the ED with shortness of breath, diffuse left-sided chest pain and weakness. After initial triage and EKG (Figure 5), she left the facility without evaluation. She returned to the ED on the following day with worsening symptoms and new subjective fever and chills. The EKG at that time showed a significant reduction in voltage and subtle PR-segment depressions in leads II, III and aVF (Figure 6). Vital signs in the ED were HR 141 BPM, BP 111/67 mmHg, RR 24 breaths per minute and SpO_2_ 100% on room air (Table 1). On examination, her extremities were cold to the touch and she was tachypneic and tachycardic without jugular venous distension. Her chest x-ray depicted a normal cardiac silhouette and did not show any pulmonary infiltrate or pleural effusions (Figure 4). The laboratory data were notable for a positive serum COVID-19 RNA PCR, mildly elevated cTnI (0.55 ng/mL), elevated serum lactic acid level (4.8 mmol/L) and unremarkable inflammatory markers (Table 1). A cardiac ultrasound demonstrated a large pericardial effusion with right ventricular diastolic collapse and severe global biventricular systolic dysfunction with an LVEF of 20%.

Ms. B became progressively hypotensive despite initial intravenous fluid administration. Emergent pericardiocentesis was performed, yielding 280 mL of serous fluid. A pulmonary arterial catheter was placed, demonstrating a PCWP of 32 mmHg and a cardiac index of 2.5 L/min/m^2^. Initially, her serum lactic acid levels normalized; however, over the following hours she developed progressive signs of cardiogenic shock. Her cardiac index declined hourly as her lactic acid increased despite escalating inotropic and vasoactive support. Considering Ms. B’s young age, isolated cardiac involvement and absence of comorbidities, support was escalated to cannulation for veno-arterial ECMO (VA-ECMO). She was treated with colchicine and intravenous pulse-dose steroids, and was successfully decannulated from VA-ECMO four days later after demonstrating modest cardiac recovery. Thereafter, she was discharged with six weeks of colchicine therapy and on follow-up her echocardiogram revealed recovery in LVEF to 60% and an absence of pericardial effusion.

## Discussion

The hospital course of these two patients with fulminant COVID-19 myocarditis who were admitted to our cardiac critical care unit within a span of four days illuminates the potential for rapid cardiac decompensation. Both patients reported viral symptoms and chest pain; however, Ms. A presented with ST-segment elevations and a highly elevated cTnI level, while Ms. B presented with a large pericardial effusion (likely of acute onset given the voltage decrement observed over 24 hours; Figure 6). They differed in age, comorbidity, ethnicity, organ system involvement, degree of cTnI elevation and inflammatory marker profile. The differences in presentation and diagnostic findings are shown in Table 1. 

While there have been reports of troponin elevation and ST-segment elevations on EKG without epicardial coronary artery disease and with non-fatal myocarditis, isolated cardiovascular involvement with fulminant myocarditis and cardiac tamponade are rarely reported complications of COVID-19 infections [4-8]. Additionally, inflammation and cytokine release through direct and indirect mechanisms have been implicated in myocardial injury; however, as reported above Ms. B continued to have inflammatory markers within normal range despite florid myopericardial disease [3,9,10]. Adding to rarity in this case, Ms. B is one of few reported cases who tolerated VA-ECMO with successful decannulation and rapid recovery in systolic cardiac function within three weeks from initial presentation [11]. This is an important finding since the role of VA-ECMO and implantable mechanical circulatory devices in COVID-19 myocardial disease has not yet been established.

## Conclusions

There is substantial variability in presentation of COVID-19 myocarditis and timely identification of this less commonly reported entity is of great importance. Treatment options for COVID-19 myocarditis are still evolving while pathogenesis remains hypothetical; however, mechanical circulatory support devices and life support therapies such as veno-venous ECMO (VV-ECMO) and VA-ECMO may be life-saving in select cases.
